# Central role of neutrophil in the pathogenesis of severe acute pancreatitis

**DOI:** 10.1111/jcmm.12639

**Published:** 2015-08-07

**Authors:** Zhi-wen Yang, Xiao-xiao Meng, Ping Xu

**Affiliations:** a Pharmacy Department, Songjiang Hospital Affiliated the First People's Hospital, Shanghai Jiao Tong UniversityShanghai, China; b Digestive Department, Songjiang Hospital Affiliated the First People's Hospital, Shanghai Jiao Tong UniversityShanghai, China

**Keywords:** severe acute pancreatitis, neutrophils, trypsin activation, NETs

## Abstract

Severe acute pancreatitis (SAP) is an acute abdominal disease with the strong systemic inflammatory response, and rapidly progresses from a local pancreatic damage into multiple organ dysfunction. For many decades, the contributions of neutrophils to the pathology of SAP were traditionally thought to be the chemokine and cytokine cascades that accompany inflammation. In this review, we focus mainly on those recently recognized aspects of neutrophils in SAP processes. First, emerging evidence suggests that therapeutic interventions targeting neutrophils significantly lower tissue damage and protect against the occurrence of pancreatitis. Second, trypsin activation promotes the initial neutrophils recruitment into local pancreas, and subsequently neutrophils infiltration in turn triggers trypsin production. Finally, neutrophils have the unique ability to release neutrophil extracellular traps even in the absence of pathogens.

## Introduction

Acute pancreatitis (AP) is a common acute abdominal disease encountered in many countries, and its incidence appears to be rising 1,2. In most cases, AP is regarded as a mild and self-limiting illness with complete *restitutio ad integrum*. Unfortunately, about 20–30% of all patients with AP eventually develop severe acute pancreatitis (SAP) in clinical practice, experiencing a severe attack with progression to multiple organ dysfunction or local complications, *e.g*. necrosis, pseudocyst or abscess 3. Despite improvements in diagnostic and therapeutic techniques, the mortality rate of SAP is up to a relatively high mortality rate ranging from 20% to 30%, without a decreasing tendency over the last decade 4,5. The emerging researches have led to considerable progress in understanding the pathophysiological process of SAP, but its underlying pathogenic mechanism is still unknown to a large extent 6. Hence, the molecular pathogenic mechanisms on SAP are of high clinical value for the effective treatment to reduce its morbidity and incidence 7.

Pancreatic enzyme autodigestion has traditionally held the spotlight for many decades as it is thought to be responsible for the onset and aggravation of pancreatitis 8,9. During an AP episode, inappropriate activation of trypsinogen inside the pancreas seems to be the direct pathogenesis in trypsin-centred pancreatitis, which leads to damaging pancreatic cell, activating pathological inflammatory signalling and triggering other cellular molecules stress responses in most clinical and experimental models 10. In fact, the usage of trypsin protease inhibitors in clinical practice fails to provide any resolution for SAP patients. Furthermore, genetic rat models specifically lacking pathological trypsin activation are successfully developed, showing little rigorous proof to establish the causality of premature trypsinogen activation and pancreatitis 11–13. Thus, recent advances pose a real challenge to understand the long-believed trypsin-centred theory of pancreatitis.

Alternatively, there is growing evidence to support the inflammatory factor theory for the crucial pathogenesis of pancreatitis, independent of trypsin activation 14. The release of inflammatory signals from acinar cells could mediate the recruitment and activation of circulating inflammatory cells, such as peripheral blood mononuclear cells and polymorphonuclear (PMN) neutrophils 15,16. Finally, excessive activation of leucocytes trigger intense local and systemic inflammatory responses, which become the hallmark of SAP responsible for high mortality and morbidity rates, *e.g*. acute respiratory distress syndrome, cardiovascular failure, renal failure and gastrointestinal haemorrhage 17.

In view that neutrophil recruitment has a central role in the onset, progression and resolution of inflammation, this review provides that the current and emerging trends of neutrophils composed of a basis for progression of SAP, highlighting some novel contributions of neutrophils to SAP processes such as therapeutic interventions targeting neutrophils, neutrophil-regulated trypsin activation and neutrophil extracellular traps (NETs) release. In the meantime, some traditional elements of neutrophil biology are omitted here, including cytokines, chemokines, signal transductions and transcellular migration.

## Neutrophil functions

Neutrophils are the terminally differentiated cells, arising from the bone marrow where they are stored and developed for release into the blood stream 18. Once released into the circulation, neutrophils could seek signs of foreign organisms and antigens and then they are recruited to sites of infection or inflammation 19. In the absence of infectious pathogen, neutrophils in the circulation have a short-lived span and they die within 24 hrs by a spontaneous apoptosis mechanism 20. When encountering inflammation signals, activated neutrophils could prolong the lifespan by several days, during which they modulate the inflammatory microenvironment, change the turnover rates of key proteins and release inflammatory mediators 21.

During persistent inflammation, activated neutrophils sequentially release some specific granule proteins, which are responsible for activating endothelial cells and inducing permeability changes in vessel walls 22. Subsequently, inflammation neutrophils are caught and migrated from the circulation into tissues through the paracellular pathways with interendothelial clefts at points where three or more endothelial cells join 23. Neutrophils across the endothelial cells aggregate around the focus of infection or injury sites, which exhibit augmented reactive oxygen species (ROS), nicotinamide adenine dinucleotide phosphate oxidase (NADPH oxidase) and granule enzymes activity, mediate neutrophil-derived chemo-attractants, contain and produce more pro-inflammatory cytokines in response to an inflammatory stimulation 24. Once neutrophil infiltration and aggregation in the tissues is overwhelming, these may fail to clear the microbial infection, impair the anti-protease and antioxidant protective reaction and lead to severe tissue damage 21,25,26.

Enhancement of neutrophil apoptosis and effective uptake of apoptotic neutrophils by macrophages abrogate further neutrophil infiltration into tissues, leading to the resolution of inflammatory response 27. As we know, apoptotic neutrophils can produce signals that operate negative feedback loops 28. Annexin A1 released by apoptotic neutrophils is the negative regulator of neutrophil influx and recruitment, resulting in dampening the accumulation of neutrophils in the tissue and preventing the release of pro-inflammatory mediators 29. In addition, neutrophils undergoing apoptosis display cell surface receptors including a tyrosine kinase receptor MeR, scavenger receptors, phosphatidylserine receptor, integrins and complement receptors, which enable their recognition and clearance by macrophages, and prevent the release of cytotoxic products from activated neutrophils into the tissue environment 20. Furthermore, uptake process of apoptotic neutrophils can stimulate macrophages to release the anti-inflammatory cytokines, transforming growth factor-β and interleukin-10 (IL-10), and suppress the inflammatory response 30,31.

Taken together, activated neutrophils hold a central position as it brings the sustained inflammatory response and tissue damage. In terms of resolution, apoptotic neutrophils are crucial for the repair process to terminate inflammation.

## Killing of pathogens

Neutrophils play a major role in host defence against the resistance to bacterial, fungal and viral infections by three distinct mechanisms, such as phagocytosis, degranulation and activation of the oxidative burst, NETs. Neutrophil-mediated phagocytosis is directly involved in the plot to kill the invading microbes. Oxidative burst in inflammatory neutrophils is capable of killing the potentially dangerous microorganisms through the release of various noxious agents (ROS and oxygen-free radicals generated through the activity of plasma-membrane-bound NADPH oxidase). Besides the well-established phagocytosis and oxidative burst, NETs are generally accepted at present. However, NETs contributing to neutrophil-mediated anti-microbial responses still have an intensive scrutiny and debate so far 32. Granule proteins, consisting of the core composition of NETs, maybe involve in the provocative molecular mechanism, because it is unclear if ion fluxes triggered by the NADPH oxidase contribute to the antibacterial function of the granule proteins. It is also debated to what extent NETs kill pathogenic microorganism and promote antibacterial host defence*,* and especially whether they can be available in sufficient quantities for activating the *in vivo* protective reaction under sterile inflammation conditions 33,34.

## Roles of neutrophils in SAP

Recent studies have found that neutrophils are central to the evolution of SAP, mediating local tissue damage in the pancreas, as well as remote organ injury and subsequent death. At the early stage of SAP, pancreatic cell damage caused trypsinogen abnormal activation-induced aseptic inflammatory signalling that recruits inflammation neutrophils into pancreas. The activated neutrophils extend the lifespan and release the high concentrations of oxidants and cytotoxic agents, which further worsen the local damage to the pancreatic tissues. As inflammation continues, the transmigration cascade of neutrophils across endothelial cells culminates. Based on a second ‘swarm’ of neutrophils, it may rapidly progress and aggravate from local pancreatic-islet inflammation into systemic inflammatory response syndrome, causing remote organ injury, multiple organ dysfunction syndrome or serious complications by overwhelming inflammatory responses.

Neutrophils from patient peripheral blood had a very different trend compared with those isolated from healthy individuals. These activated neutrophils significantly extended the lifespan and functional activity, which maybe contribute to pro-inflammatory cytokines secretion, cell migration and invasion, and neutrophil apoptosis delay 35–37. Thus, the induction of neutrophil apoptosis can facilitate the resolution of inflammation by recovering the neutrophil lifespan *in vivo*. The mechanisms responsible for activated neutrophils in local pancreatic inflammation include cytokine-mediated changes in gene expression, complement activation in plasma and alterations in the turnover rates of key proteins 38,39. For example, tumour necrosis factor-α (TNF-α), IL-1 and IL-6 have been postulated to promote neutrophils adherence and extravasation, increase capillary permeability, aggravate pancreatic injury, as well as systemic inflammatory response syndrome 40–42. A second mechanism of neutrophil activation in peripheral blood is involved in the release of the anaphylatoxins C3a and C5a complement in plasma, which may enhance activation and infiltration of inflammation neutrophils leading to vascular leakage 43. In addition, immune complexes, *e.g*. iC3b complement covalently deposited on the surface of endothelial cell membranes, can act as a signal transuding partner for neutrophils to undergo an oxidative burst *via* the integrin CD18 and CD11b 44. After the rolling and activated neutrophils attach to the vessel wall and then exit the circulation into tissues, some signalling mediated by neutrophil-derived chemo-attractants, such as leukotriene B4 and G protein-coupled receptors, makes possible the long-distance migration of neutrophils in the vital tissues 45. Although neutrophils recruited were known as a non-specific defence reaction against the invading microbes, excessive recruitment and activation of neutrophils can lead to the presence of extensive organ dysfunction along with massive pro-inflammatory mediators and reactive oxygen intermediates. As a result, patients with SAP usually die of multi-organ function failure through the development and progression of systemic inflammatory cascade-induced pancreatic injury, not through the pancreatic tissue damage itself 46. Almost 60% of SAP deaths occur within the first 7–14 days, associating with acute lung injury 47.

Traditionally, the main contribution of neutrophils to the central pathogenic event of pancreatitis was traditionally thought to be their release of some inflammation products for many years. However, recent experimental evidence strongly suggested that neutrophils also had an active role in orchestrating the progress of pancreatitis, through regulating neutrophil-regulated trypsin activation and NETs release. These advances in the field would further enrich the existing neutrophil-centred theory of pancreatitis.

## Therapeutic interventions targeting neutrophils

Numerous studies had demonstrated that the systemic depletion of neutrophils or the secretion of activated neutrophils significantly lowered tissue damage and protected against pancreatitis. Thereby, some interventions targeting neutrophils were used to treat SAP in practice, through inducing neutrophil apoptosis and decreasing ROS production, degranulation, leukotriene B_4_ synthesis and neutrophil migration, and so on 48.

Anti-rat neutrophil antibody (PoAb) to deplete peripheral neutrophil counts was used to assess the neutropaenia effects in a rat model of acute necrotizing pancreatitis. The administration of PoAb was an effective therapy for preventing acute lung injury caused by SAP 49. Lung myeloperoxidase (MPO) activity and histopathology indicated that neutrophil infiltration into the lung was obviously diminished by anti-neutrophil antibody. Urge-8 antibody, a mouse monoclonal antibody to neutrophils, induced selective depletion of circulating neutrophils *in vivo*. They were used to treat acute necrotizing pancreatitis and its related symptoms to rats. The results suggested that Urge-8 antibody to SAP rats could obviously prolong the survival time and reduce the failure in the vital signs, such as mean arterial pressure, heart rate and body temperature 50.

Intercellular adhesion molecule (ICAM) was known to mediate the adherence of neutrophils to endothelium. In this study, ICAM-1 antibody could obviously inhibit neutrophil adherence and activation, lower O_2_^−^ production secreted by neutrophils, prolong rat survival and attenuate pulmonary inflammatory responses in the rat model of AP 51. Anti-rat monoclonal antibody (MoAb) CD18 to block neutrophil adherence functions was used to block CD18-dependent neutrophil adhesion in rats. The results indicated it was an effective treatment for acute lung injury during the neutropaenia 49.

Cytokine-induced neutrophil chemoattractant (CINC), the rat homologue of human growth-regulated oncogene-α (GRO-α), was a C-x-C chemokine with specific neutrophil activator and chemoattractant 52. CINC was correlated with disease severity of SAP through the induction of neutrophil recruitment *in vivo* and specific antagonists to inflammatory response 53. In this study, anti-CINC neutralizing antibody was applied to examine the therapeutic effects on pancreatic and lung damage using a model of pancreatitis rats induced by caerulein. Anti-CINC neutralizing antibody groups did not alleviate the local pancreatic damage along with an increase in plasma amylase and pancreatic oedema, but they showed a significant protection against SAP associated lung injury. The study suggested that anti-CINC neutralizing antibody would not block neutrophil recruitment, activation, infiltration and migration in pancreatitis, which was consistent with the previous reports that neutrophil-mediated damage mainly occurred in some vital organs (*e.g*. lung, kidney, liver), but not in pancreatic tissues 54,55.

Tumour necrosis factor α, a potential and selective activator of neutrophils *in vitro and in vivo*, appeared to be an important inflammatory mediator in the occurrence and progress of SAP. High level of TNF-α in patients could induce pancreatic and other organs' damages, which directly led to the mortality and severity of the disease 56. Recent articles further supported that TNF-α antibody played an important protective role in pancreatitis in rats. TNF-α antibody could effectively ameliorate the selected biochemical parameters of severe pancreatitis, block the TNF-induced phagocytic cell activity, ablate the TNF-dependent feedback loops that generate abundant pro-inflammatory cytokines, inhibit the endogenous antioxidant activity, lower the microvascular permeability, decrease the concentrations of matrix metalloproteinase (MMPs) and influx of inflammatory neutrophils by down-regulating adhesion molecule expression, and induce neutrophil apoptosis 57,58. In contrast to the expected findings, another study found that the blockade of serum TNF-α increased the formation of pulmonary and pancreatic oedema in microvascular beds, indicating little therapeutic effect of TNF-α antibody in SAP 59. The reason for these discrepant results was not known to date. Thus, TNF-α antibody influence on inflammatory response still need further investigation in the future.

Interleukin 8 (IL-8), one of the key pro-inflammatory cytokines involved in the propagation of AP from the localized inflammatory and haemorrhagic necrosis into systematic inflammatory response syndrome, had been regarded as a neutrophil chemotactic and activating cytokine. Furthermore, serum IL-8 levels showed a positive correlation with its systemic complications of SAP 60,61. Monoclonal anti-human IL-8 antibody to rabbits with SAP could attenuate the inflammatory response in lung tissue and decrease the severity of pancreatitis, through the inhibition of circulating pro-inflammatory cytokines IL-8 and TNF-α, removal of the neutrophil infiltration into the pancreas and lung tissue and down-regulation of the adhesion molecule complex CD11b/CD18 62.

As a result of the absence of appropriate genetic tools to specifically delete neutrophils, we do not provide the strong evidence that neutrophil activation is the pathophysiological hallmark of AP responsible for high mortality and morbidity rates. For example, gene knockout mice (such as lacking granule proteins expressed in neutrophils) were obtained to examine the neutrophil-centred theory of pancreatitis. However, these proteins and genes were also expressed in other myeloid cells, not to allow exclusion of an effect on other leucocyte lineages. In addition, various antibodies to deplete neutrophils may also deplete leucocyte lineages other than neutrophils 63.

## Neutrophils in the activation of trypsinogen

As reported previously in the literature, neutrophil infiltration and trypsinogen activation were considered to play a pivotal role in the pathophysiology of SAP. Both of them constituted the hallmarks of SAP, leading to local complications and systemic inflammation 46. However, few authors had attempted to cover their inter-relationships in mediating the inflammatory process in pancreatology. Here, we pay more attention to the intimate partnership of neutrophils and trypsinogen in SAP, suggesting that trypsinogen activation induces initial neutrophil infiltration into local pancreas and subsequently neutrophil infiltration in turn triggers trypsinogen production.

It was widely held that the activation of trypsinogen to trypsin regulates neutrophil infiltration into pancreas 64. Specific adhesion molecules and pro-inflammatory cytokines, such as P-selectin 65, lymphocyte function antigen-1 (LFA-1) 66, NF-κB, TNF-α and IL-6, were released from acinar cells undergoing oedema and necrosis after autodigestion, which also promoted the recruitment of neutrophils into pancreas. Direct evidence had presented that activation of trypsinogen to trypsin induces neutrophil infiltration into pancreas through genetic knockout models. Inhibition of trypsin activation by deleting trypsinogen-7 or cathepsin B gene had shown that it was important to reduce pancreatic damage along with a reduction in neutrophil infiltration. Trypsinogen-7 knockout mice, genetic models specifically lacking pathologic trypsinogen activation, suggested about 50% reduction in acinar necrosis and similar reduction tendency in neutrophil infiltration into pancreatic tissue during the early stages of pancreatitis 10. Similar results in cathepsin B^−/−^ mice had been found, which was responsible for reducing pancreatic damage indicated by serum levels of amylase and histomorphology of pancreatic tissues 13. In addition, cathepsin B inhibitors contributed to retard trypsin activation, showing a protective role during pancreatitis by reducing pancreatic injury and neutrophil infiltration 67,68.

Subsequently, neutrophils recruited into pancreatic tissues in turn contributed to trypsin activation in pancreatic acinar cells during pancreatitis process. Trypsinogen activation peptide (TAP) was formed in equimolar amounts during conversion of trypsinogen to trypsin, which was used as a marker of trypsin activation 69. Significantly increased concentrations of TAP were found by incubating the activated neutrophils with trypsinogen for 20 min. at room temperature, demonstrating that inflammatory neutrophils could promote trypsinogen to trypsin *in vitro* 70. To our knowledge, this observation was the first to be reported, and the role of circulating neutrophils in trypsin activation and its molecular mechanism remained unclear 70. To further investigate the role of activated neutrophils in trypsin activation, activated neutrophils or secretions from activated neutrophils were respectively incubated with acinar cells *in vitro*. Coincubation of activated neutrophils or its secretions significantly stimulated trypsin activation in acinar cells. What is more, trypsin activation in acinar cells was up to 1.72 times by incubating with neutrophil secretions. In the *in vivo* study, change in TAP levels was correlated with neutrophil depletion in rats with taurocholate-induced pancreatitis, showing that the initial elevation of TAP levels was insensitive to neutrophil depletion within 2 hrs after taurocholate challenge while TAP levels markedly reduced at 24 hrs as a result of neutrophil depletion 71. Based on these data, it had direct evidence that trypsin activation in the pancreas was a dynamic process requiring inflammatory neutrophils, which was characterized by an early neutrophil-independent and a late neutrophil-dependent phase. In a word, the *in vivo* and *in vitro* results supported the conclusion that circulating neutrophils could stimulate trypsin activation in the pancreatitis and aggravate pancreatic damage 71.

Matrix metalloproteinase-9, an abundant protease mainly located on the secretory granules in neutrophils, was forwarded as a potential prognostic marker in pancreatitis 72,73. This might also represent an efficient tool for investigating the mechanism of trypsin activation induced by neutrophils. Inhibitor studies, including a broad-spectrum inhibitor of neutrophil-derived MMPs and MMP-9 gene-deficient mice, were used to evaluate trypsin activation in taurocholate-induced pancreatitis 74. First, the *in vivo* studies were conducted in the following article. BB-94 treatment group, a broad-spectrum inhibitor of MMPs, markedly reduced pancreatic TAP formation by 61% and decreased acinar cell necrosis by 69% in mice. MMP-9-deficient mice exhibited that taurocholate-induced activation of trypsin and neutrophil infiltration was markedly attenuated in the pancreas. Second, the *in vitro* experiments were conducted to assure the role of neutrophils and MMP-9 in trypsin activation through the incubation of isolated pancreatic acinar cells with neutrophils or secretions from neutrophils. It was found that stimulation of acinar cells with activated neutrophils had positive effect on trypsin activation in acinar cells, while activated neutrophils from MMP-9 gene-deficient mice did not activate trypsinogen to trypsin in acinar cells. Moreover, trypsin activation in acinar cells was up to 162% after the incubation of acinar cells with secretions from activated neutrophils. In contrast, trypsinogen activation in acinar cells was insensitive to the stimulation when coincubated with secretions from MMP-9-deficient neutrophils. In addition, activated rMMP-9 can effectively convert trypsinogen to trypsin in acinar cells. Thus, neutrophil-derived MMP-9 as an important regulator was responsible for neutrophil-dependent trypsin activation.

Another experiment demonstrated that anti-neutrophil serum to rats with cerulein-induced pancreatitis markedly attenuated trypsin activation in pancreas and acinar cells, associated with severe depletion of circulating neutrophils. To example the possible mechanisms, NADPH oxidase gene-deficient mice were carried to localize intrapancreatic activation of trypsin within the acinar cell. Compared with controls, mice deficient in NADPH oxidase showed the poor intrapancreatic trypsin activation and low acinar TAP levels induced by high-dose cerulein. These findings revealed that neutrophil-dependent trypsin activation could be abolished in the absence of NADPH oxidase in neutrophils 75.

However, it seemed to be an independent pre-condition for trypsin activation *via* neutrophil infiltration into the pancreas in several studies. LFA-1 on the surface of circulating neutrophils played an important role in neutrophil adhesion and infiltration into the pancreas. Interference with LFA-1, an antibody against LFA-1 or LFA-1 gene-deficient mice, did not alter taurocholate-induced trypsin activation in the pancreas. The similar results were found in Toll-like receptor 4 (TLR4)-deficient mice 76. Thus, accumulating data in these literatures supported that intrapancreatic trypsin activation was independent of neutrophil-mediated pathway 66.

In summer, the inter-relationships of neutrophils and trypsinogen are some of the most pressing issues in current SAP research. The mechanism that underlies intra-acinar activation of digestive enzymes (trypsin) still remains unclear.

## Neutrophil extracellular traps

A study in 2004 first reported that neutrophils had a hitherto unrecognized mechanism of inflammation and autoimmune diseases, in which neutrophils could release NETs to trap and kill extracellular microorganisms 77. As we know, NETs had been found to be abundant against the invading microbes during the *in vivo* infection 78,79. Through NETs formation, neutrophils provided the complex of neutrophil granules and histones with powerful anti-microbial activity. When NETs formation was blocked, it would fail to clear microbial infections *in vivo* 80. Thus, NETs played a protective role against microorganisms, prevented microbial dissemination to distant organs and ensured a relatively high local concentration of anti-microbial agents to kill bacteria and degrade virulence factors 80.

Neutrophil extracellular traps consisted of chromatin bound to granular and nucleic proteins, characterized by smooth filaments with a diameter of 17 nm and studded with globular domains made of granular proteins 81. Fully hydrated NETs with a cloud−like appearance were found using high resolution scanning electron microscopy, and NETs volume was about 10- to 15-fold bigger than a native human neutrophil 82. Different from the previously reported neutrophil apoptosis and necrosis, the release of NETs offered an alternative to cellular death that expelled their chromatin and granule proteins for controlling microbial infections at the end of the lifespan. NETs formation could induce the dramatic change in the nucleus of neutrophils, undergoing the process of nuclear envelope breakdown, chromatin expansion and decondensation. Accompanied by disintegration of neutrophil granules, morphological chromatin bound to granular proteins culminated within cells. Finally, cell membrane ruptured, resulting in NETs was released from the intracellular to the extracellular space.

Lots of research had been done to identify the molecular mechanisms that regulated NETs formation. For example, ROS induced *via* neutrophil NADPH oxidase was tightly linked to NETs formation. As a result, chronic granulomatous patients with mutations in NADPH oxidase enzyme were incapable of forming ROS, leading to the absence of NETs formation 83. In agreement with this requirement, individuals with MPO deficiency, another key enzyme involved in the ROS production, also fail to make NETs. Furthermore, phorbol myristate acetate (PMA), the most potent inducer of NADPH oxidase, was carried to confirm the NETs formation in PMA-stimulated neutrophils. Upon neutrophil activation, kinases Raf, Mitogen-activated extracellular signal-regulated kinase (MEK) and extracelular signal-regulated kinase (ErK) pathway was involved in NETs formation through upstream of NADPH oxidase but downstream of protein kinase C (PKC) 84–86. Because the formation of ROS was catalyzed by NADPH oxidase and MPO, ROS production played a vital role in NETs formation. Although important, ROS may be not the only crucial players to form NETs formation in the presence of some neutrophil stimuli 87,88. Recently, a second mechanism related to peptidylarginine deiminase 4 (PAD4) was found to form NETs *in vitro and in vivo*. PAD4, a critical protein citrullinating enzyme that mediated chromatin decondensation, was shown to enter the nucleus for degrading histones. Upon histones were cleavages, which led to relaxation and decondensation of chromatin, and finally produced NETs 89,90. In the cellular model of PAD4 overexpression, activation of PAD4 was identified as the primary driving force, resulting in chromatin decondensation and NETs release 91. On the contrary, NETs formation *in vivo* was completely inhibited In PAD4-null mice 92,93.

There had been a large number of studies that proposed some interesting roles for NETs in inflammation, autoimmunity and vascular diseases. However, there was an ongoing debate whether NETs may contribute to some non-infectious diseases under the sterile condition without extraneous stimulation. In this section, we would focus on the contribution of NETs to aseptic pancreatitis.

Acute pancreatitis is characterized by non-infectious inflammation without fungal and bacterial antigens recognition. To our knowledge, an endogenous substance from the human body itself might ignite inflammation response of AP patients. During the inflammation process, activated neutrophils leak out ROS, NADPH oxidase and granule enzymes. This is responsible for the key mechanism and core composition of NETs formation. Insights from the *in vivo and in vitro* models about the presence of NETs in pancreatitis were first reported in this study 94. First, pancreatic acinar cell injury by histones (as the constituent of NETs) was investigated *in vitro*. Pancreatic acinar cells were incubated with histones at the concentration of 0, 50, 100 and 200 mg/ml. Necrosis in acinar cells peaked at 60 min., and the necrosis rate at 60 min. was a dose-dependent increase under stimulation by histones. Second, NETs was induced *via* PMN cells stimulated with phorbol 12-myristate 13-acetate. After incubation with NETs, mean necrosis in acinar cells also increased in response to PMN-extracted NETs but not heat-inactivated NETs. Finally, C57Bl/6J mice with palmitoleic acid + ethanol-induced AP were carried to study whether NETs can indeed be generated *in vivo*, and when they were formed. NETs formation was found in serum and pancreas at 12 hrs, and appeared to be the peritubular distribution, coinciding with time and areas of pancreatic necrosis. Taken together, NETs and their constituent components caused necrosis in freshly isolated pancreatic acinar cells and were detectable in an *in vivo* model of AP only within necrotic segments of the pancreas. The study was the first to raise interesting novel roles for NETs in pancreatitis without microbial infection.

## Conclusion

The coordinated interplay between neutrophils and trypsinogen is crucial for the control initiation, progression and resolution of inflammation in pancreatic acinar cells. In addition, the discovery that NET formation is associated with pancreatic necrosis, opens up a new avenue for understanding the role of neutrophils in this disease. These advances about neutrophil function in the progression of pancreatitis are leading directly to an interesting strategy to eliminate noxious agents and restore tissue homoeostasis.
